# Private Nursing under a State Service

**Published:** 1920-11-06

**Authors:** 


### Private Nursing under a State Service.

A good many nurses are frankly captivated by the notion of a State Nursing Service propounded by "Ierne." It seems to promise in a golden haze relief to the hospitals from the burden of maintaining the training-schools, relief to the public who are to get their nursing for nothing, and splendid salaries to the chosen band. We need not dwell on the colossal cost of this suggested State Service. A time may come when the public prefers to pay out, through the medium of rates or taxes, the major part of their income to enable the State to discharge for them the obligations which now periodically deplete their purses. But the objection we foresee is the repugnance of patients to submit themselves to the "State nurse." Even at the present time, under a subscription of some 2s. 6d. a year or none at all, patients are found recalcitrant to the ministrations of district nurses who do not take their fancy. Would not the very notion that the nurse was foisted upon them from above multiply a thousand-fold those drastic criticisms of private nurses with which we are all familiar?

Truth to tell, we are not by any means sure that to belong to a "State service" does add so enormously to the prestige of the individual. We seem to remember pretty widespread discontent on the part of elementary school-teachers and a corresponding lack of candidates for this service. The junior ranks of the Civil Service are by no means remarkable for their appreciation of the ægis conferred by serving the State, nor is promotion either rapid or certain. To rise out of the dead level of mediocrity is always more difficult the more highly organised the profession becomes. Individual control affords numerous opportunities and by-paths to affluence which would be denied to nurses under State control, though a tolerable number of supervising posts would be created. For every nursing sister rejoicing in the prizes offered in "the uplifted nursing world," it is manifest there must be a hundred or so grumbling at the lack of prospects.

In one point we are heartily in agreement with the notion of a State nursing service. It would introduce that universal and non-voluntary pension scheme after a given number of years' service, which is the great need of nurses at the moment. Perhaps nothing short of some mild State control could bring us this.

